# Disease Burden on Health Facilities in Governorates South of Karbala During the Arbaeenia Mass Gathering in Iraq in 2014: Cross-Sectional Study

**DOI:** 10.2196/10917

**Published:** 2019-10-16

**Authors:** Hayder Hantoosh, Faris Lami, Basel Saber

**Affiliations:** 1 Thiqar Directorate of Health Iraq Ministry of Health Thiqar Iraq; 2 Department of Community and Family Medicine College of Medicine University of Baghdad Baghdad Iraq; 3 Muthana Directorate of Health Iraq Ministry of Health Muthana Iraq

**Keywords:** mass gathering, Arbaeenia, Iraq, disease burden

## Abstract

**Background:**

Millions of Iraqi pilgrims travel annually from the southern governorates to Karbala and pass through Thiqar, Muthana, and Diwania Governorates to join the Arbaeenia mass gathering event. During this event, participants are at high risk for diseases and death and stifle local health care resources. In addition, the mass gathering causes considerable burden on health facilities in the hosting localities.

**Objective:**

This study aims to estimate the disease burden on health facilities caused by the pilgrims passing through Thiqar, Muthana, and Diwania Governorates en route to Karbala in Iraq.

**Methods:**

This cross-sectional study was conducted on all health facilities in three governorates (Thiqar, Muthana, and Diwania) situated along the southern way to Karbala from Basra. The study started on December 11, 2014, and ended on December 24, 2014. The morbidity and mortality were collected from surveillance logbooks and death registers. Drug purchase data were obtained from the personnel in charge of the pharmacies. The study period was divided into three phases on the basis of the timing of the mass gathering event: pre-event, the event, and postevent.

**Results:**

There were 884,834 incidents reported during the study. The majority of incidents were reported during the event phase (95%) and were attended mostly at mobile clinics (77%). The average daily incidents during the pre-event, event, and postevent phases were 4300, 56,040, and 4548 incidents, respectively. Musculoskeletal disorders were the most common illness reported (55%). The average number of daily deaths was 43, 36, and 45 during the pre-event, event, and postevent, respectively, and these values did not differ significantly. Cardiovascular diseases (43.5%), injuries (29.8%), and respiratory illnesses (12%) were the leading causes of deaths. Approximately US $1.3 million was spent on drug purchases during this mass gathering in the three governorates.

**Conclusions:**

The Arbaeenia mass gathering causes a tremendous disease and economic burden on governorates that pilgrims pass through to attend this mass gathering in Karbala. Although Iraq’s Ministry of Health is aware of the high burden of this mass gathering on the health facilities in these governorates, more work is needed to ensure quality services during the event.

## Introduction

The World Health Organization defines mass gatherings as “events attended by a sufficient number of people to strain the planning and response recourses of a community, state or nation” [1,2]. Mass gatherings are one of the most signiﬁcant items on the global health security agenda [3] and can occur spontaneously without previous alerts, such as political rallies, protests, or planned gatherings. They can be either periodic at different localities like the Olympics and World Soccer Cup or repeated events at the same place like the Hajj in Saudi Arabia, which is one of the largest mass gathering worldwide [4].

Studies on mass gatherings have concluded that the event type, duration, attendance, weather, mood, density, and other factors determine the medical services needed. Mass gatherings are a challenge to public health authorities and pose a significant burden for the preparedness and response to potential mass causalities and disease outbreaks [5]. Besides the risk of acquiring different infectious diseases, mass gathering attendees are at an increased risk for injuries that range from mild muscle cramps to severe injuries and exacerbation of pre-existing noncommunicable disease conditions that can be fatal [6-8].

**Figure 1:**
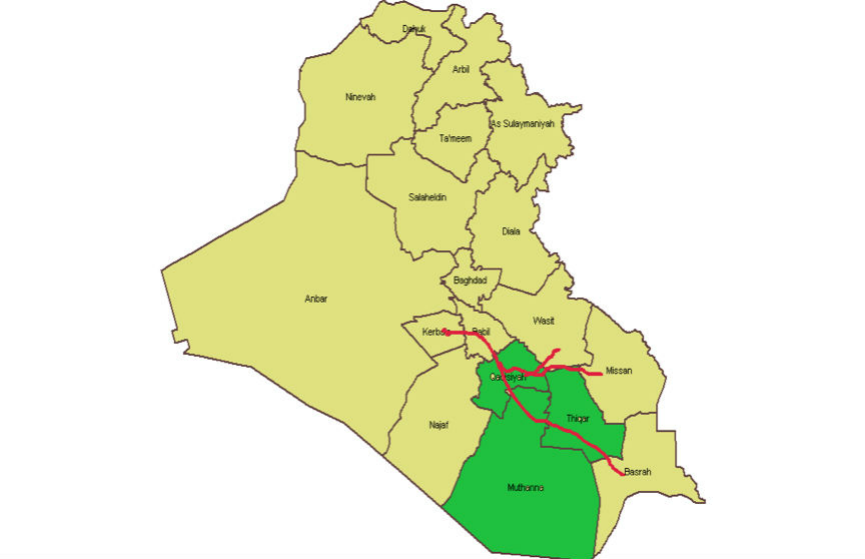
Iraq map and the three studied governorates.

Many religious mass gathering events take place in Iraq annually, mainly in Karbala, Najaf, and Baghdad [9]. The Arbaeenia mass gathering attracts millions of local and international Shiite Muslim pilgrims [10]. Around 3 million pilgrims travel on foot from Basra, Thiqar, Muthana, Diwania, and Babel Governorates to Karbala to join the Arbaeenia mass gathering event [11]. The distance from Basra to Karbala is about 600 km and passes through four governorates (Figure 1).

There have been a few studies on the burden of religious mass gatherings on Iraq’s local health resources [12]. This study was conducted to estimate the burden of diseases on local health facilities along the way from Basra to Karbala during the Arbaeenia mass gathering. The Ministry of Health and local health directorates can utilize the data from this study to develop plans and strategies for logistics, plan financing, and provide technical support to the governorates during future mass gatherings.

## Methods

We conducted this cross-sectional study on all health facilities including major referral hospitals, primary health care centers and units, faith-based health outlets, government mobile clinics, forensic units, and drugstores located along the road from Basra to Karbala. The selected facilities were limited to three governorates: Thiqar, Muthana, and Diwania. The surface area of Thiqar, Muthana, and Diwaniya governorates is 12,900, 19,980, and 8153 km^2^, respectively, and their population is 2.8, 0.87, and 1.25 million, respectively. The study started on December 11, 2014, and ended on December 24, 2014. In Iraq, this is wintertime, and the weather, in general, is cold and dry with occasional rains. The data were collected in three phases: before the mass gathering event (pre-event), during the mass gathering event (event), and after the mass gathering event (postevent). The duration of data collection for the pre-event, event, and postevent phases was 5 days, 15 days, and 5 days, respectively.

The available surveillance system data were used for the study. Data were collected on health events from seven disease categories—cardiovascular, respiratory, musculoskeletal, gastrointestinal tract, central nervous system (including headache), injuries, and other disorders—were abstracted from surveillance logbooks and forms on a daily basis by using predesigned forms. Deaths from the seven disease categories were abstracted from death registers and coroner offices. Cost data on purchases of antibiotics, analgesics, antihistamines, antispasmodic, cough syrups, ointments, and others during the event phase were collected through direct interviews with the personnel in charge of the pharmacies in these facilities.

We used Epi Info 7 software (Centers for Disease Control and Prevention, Atlanta Georgia) for data entry and analysis. Data were entered daily and cleaned and edited before analysis. We calculated the means, frequencies, and percentages of disease categories and drug expenditures by study phase: pre-event, event, and postevent. The Chi-square test was used to test significant associations among categorical variables at *P*<.05, and the Fisher exact test was used when the Chi-square test was not applicable.

## Results

There were 884,834 health incidents attended to at the selected health facilities in the three selected governorates during the study period. The majority were addressed in Diwania governorate health facilities (54.8%), and around 22% and 23% were addressed in the health facilities of Thiqar and Muthana Governorates, respectively. The majority (95%) attended the health facilities during the mass gathering event; 2%, in the pre-event; and 3%, in the postevent (Table 1). Musculoskeletal disorders were the most common disorder reported (55.2%), and injuries were the least commonly reported (3%). Cardiovascular disorders, gastrointestinal illnesses, and respiratory disease illnesses accounted for approximately 9% of the reported diseases (Table 1). The majority of the diseases (77%) were served at mobile clinics and 4.3% were served at hospitals. The disease categories varied significantly among the pre-event, event, and postevent periods (*P<*.001).

**Table 1:** Distribution of attendees to health facilities by disease categories, governorate, type of health facilities, and event phases.

Factors		Pre-event, n (%)	Event, n (%)	Postevent, n (%)	Total, n (%)	*P* value
**Disease category**						<.001
	Cardiovascular disorders	3246 (15.1)	73,479 (8.7)	3670 (16.1)	80,395 (9.1)	
	Central nervous system disorder	1453 (6.8)	33,083 (3.9)	786 (3.5)	35,322 (4.0)
	Gastrointestinal disorder	3143 (14.6)	72,773 (8.7)	2723 (12.0)	78,639 (8.9)
	Injuries	645 (3.0)	23,392 (2.8)	2145 (9.4)	26,182 (3.0)
	Musculoskeletal	4793 (22.3)	478,929 (57.0)	4583 (20.2)	488,305 (55.2)
	Respiratory disorders	4344 (20.2)	64,575 (7.7)	5919 (26.0)	74,838 (8.5)
	Others	3876 (18.0)	94,362 (11.2)	2915 (12.8)	101,153 (11.4)
**Governorate**						<.001
	Thiqar	11,180 (52.0)	178,206 (21.2)	7732 (34.0)	197,117 (22.3)	
	Muthana	4300 (20.0)	194,177 (23.1)	4071 (17.9)	202,548 (22.9)
	Diwania	6020 (28.0)	468,210 (55.7)	10,938 (48.1)	485,169 (54.8)
**Type of health facility**						<.001
	Mobile clinic or unit	0 (0.0)	682,562 (81.2)	0 (0.0)	682,562 (77.1)	
	Public health centers	17,683 (82.2)	129,086 (15.4)	17,090 (75.2)	163,859 (18.5)
	Public hospital	3817 (17.8)	28,945 (3.4)	5651 (24.8)	38,413 (4.3)
	Total number of incidents	21,500 (2.4)	840,593 (95.0)	22,741 (2.6)	884,834 (100.0)	

The daily average of events attended in the health facilities increased by 13 times during the mass gathering event compared to pre-event, of which musculoskeletal disorders showed the highest increase (33 times) followed by injuries (12 times). The average daily attendance varied among the pre-event, event, and postevent periods in disease categories (*P*=.001; Table 2).

**Table 2:** Average daily incidents attended by disease categories, governorates, type of health facilities, and event phase.

Factors		Pre-event, n (%)	Event, n (%)	Postevent, n (%)	*P* value
**Disease category**					<.001
	Cardiovascular disorders	649 (15.1)	4899 (8.7)	734 (16.1)	
	Central nervous system disorder	291 (6.8)	2206 (3.9)	157 (3.5)	
	Gastrointestinal disorder	629 (14.6)	4852 (8.7)	545 (12.0)	
	Injuries	129 (3.0)	1559 (2.8)	429 (9.4)	
	Musculoskeletal	959 (22.3)	31,929 (57.0)	917 (20.2)	
	Respiratory disorders	869 (20.2)	4305 (7.7)	1184 (26.0)	
	Others	775 (18.0)	6291 (11.2)	583 (12.8)	
**Governorates**					<.001
	Muthana	2,236 (52.0)	11,880 (21.2)	1546 (34.0)	
	Thiqar	860 (20.0)	12,945 (23.1)	814 (17.9)	
	Diwania	1204 (28.0)	31,214 (55.7)	2188 (48.1)	
**Type health facility**					<.001
	Mobile clinic or unit	0 (0.0)	45,784 (81.7)	0 (0.0)	
	Public health center	1806 (42.0)	6501 (11.6)	3130 (68.8)	
	Public hospital	2494 (58.0)	3755 (6.7)	1418 (31.2)	
	Total daily average incidents	4300 (100.0)	56,040 (100.0)	4548 (100.0)	

During the study period, 975 deaths were reported: 210 pre-event, 540 during the event, and 225 postevent. Cardiovascular diseases, injuries, and respiratory illnesses were the leading causes of death (43.5%, 29.8%, and 12%, respectively; Table 3). The average number of daily deaths in the pre-event phase was 43; 36 deaths were reported during the event, and 45 deaths were reported in the postevent phase. The number of daily average deaths did not vary between the pre-event, event, and postevent periods according to the disease categories.

**Table 3:** Distribution of average daily deaths by cause of death and event phase.

Disease category	Pre-event, n (%)	Event, n (%)	Postevent, n (%)	Total, n (%)
Cardiovascular disorders	19 (35.6)	17 (31.0)	18 (33.3)	54 (43.5)
Central nervous system disorder	2 (42.3)	1 (19.2)	2 (38.5)	5 (4.0)
Gastrointestinal disorder	0 (0.0)	1 (50.0)	1 (50.0)	2 (1.6)
Injuries	11 (29.9)	11 (30.4)	15 (39.7)	37 (29.8)
Musculoskeletal	0 (0.0)	0 (0.0)	0 (0.0)	0 (0.0)
Respiratory disorders	6 (37.4)	4 (28.0)	5 (34.8)	15 (12.1)
Others	4 (37.7)	2 (22.6)	4 (39.6)	11 (8.9)
Average daily death	42 (34.4)	36 (29.3)	45 (36.3)	124 (100.0)

Around US $1.3 million was spent on drug purchases for the mass gathering attendees during this Arbaeenia mass gathering. The highest amount spent on drug purchases was recorded in Muthana Governorate (US $568,333), and the least spent on drug purchases was in Thiqar (US $243,750) Governorate during this mass gathering. Analgesics and antibiotics were on the top of the cost of drug purchased during this mass gathering (US $315,500 and US $242,500, respectively). The least amount of money spent on purchased drug was for antihistamines (US $95,833; Table 4).

**Table 4:** Drug purchases (US $) during the Arbaeenia mass gathering.

Drug items	Governorate			
	Thiqar	Muthana	Diwania	Total
Antibiotics	59,250	91,667	91,583	242,500
Analgesics	60,583	170,833	84,083	315,499
Antihistamines	11,917	12,500	71,583	96,000
Antispasmodic	30,500	136,667	51,583	218,750
Cough syrups	12,167	89,167	52,167	153,501
Ointments	41,417	20,000	92,417	153,834
Others	27,917	47,500	50,750	126,167
Total	243,750	568,333	494,167	1,306,250

## Discussion

The Arbaeenia mass gathering puts a tremendous onus on local health resources. This study demonstrates the extra burden on the health facilities in the three governorates: Muthana, Thiqar, and Diwania. In the pre-event phase, the health attendance was maximum in Thiqar, lower in Diwaniya, and lowest in Muthana. This is a clear reflection of the total population and hence the total attendees at the health outlets.

During the event and postevent phases, the maximum number of attendees was noted in Diwaniya. As demonstrated in the map, Diwaniya is the center of cumulation of attendees coming from the southern governorates, besides the attendees coming through the second southern route from Wasit and Misan Governorates.

There was a 13-fold increase in the number of health incidents addressed the health facilities during the mass gathering event, but the daily average number of deaths during the mass gathering event did not differ from that of the pre-event. The leading causes of deaths were cardiovascular diseases, injuries, and respiratory diseases. Musculoskeletal ailments and injuries were attributed to the increased burden of health events managed at the clinics and hospitals.

The drug expenses incurred during the event were mostly from analgesics and antibiotics. A study by Chang et al [13] shows findings similar to those of this study. These medications are a suitable treatment for the common ailments experienced by the masses walking to Karbala. The journey to Karbala requires several days of walking, resulting in musculoskeletal pain and injuries, which explain the large amount of money spent on analgesics for controlling muscular pain. In addition, a considerable amount of money was used for purchasing antibiotics that mostly attributed to the frequently reported respiratory infection. Moreover, it is worth mentioning that prescribing antibiotics in Iraq, particularly during such an event, did not follow the standards of antibiotics prescription.

The health service burden during such events falls mainly on mobile clinics, where the cases are managed. This could be due to the high occurrence of minor illnesses during mass gathering as well as the accessibility of mobile clinics to the mass gathering participants. The burden on mobile clinics is consistent with that reported in a study from Australia, which shows that only one-fifth of mass gathering attendees are transferred to the hospital [14].

Intense physical activities during mass gatherings, such as walking long distances, could exacerbate cardiovascular diseases and may lead to sudden death. A study during the Hajj mass gathering indicated that cardiovascular disease is one of the leading causes of deaths [15], which was also observed in this study. Injuries were the least reported events but the second leading cause of death, which may indicate that a substantial portion of injuries were fatal.

We can conclude that the Arbaeenia mass gatherings caused a tremendous disease and economic burden to the governorates that the pilgrims pass through. In spite of the high increase in morbidity, there was no change in the pattern of mortality throughout the study period. In spite of the tremendous work done by Iraq Ministry of Health and faith-based organizations to serve pilgrims during mass gatherings, more work is needed to mitigate the burden on the health facilities in all governorates in charge of serving the pilgrims. This should be considered while developing a preparedness plan for the coming mass gatherings.

The study has certain limitations. The study covered only three governorates, which limits generalization of the study findings to other governorates and to Iraq. In addition, the data do not provide all the calculation of rates of event occurrence. Furthermore, information on health care resources in the three governorates was not available to assess the adequacy to manage the increased disease events. Finally, since most of the health outlets were concerned with provision of ambulatory health services, detailed data on risk factors and diseases were not collected.
